# Amyloid β,α-Synuclein and Amyloid β-α-Synuclein Combination Exert Significant but Different Alterations in Inflammatory Response Profile in Differentiated Human SH-SY5Y Cells

**DOI:** 10.1021/acsomega.3c05585

**Published:** 2023-11-22

**Authors:** Ebru Keskin, Duygu Gezen-Ak, Erdinç Dursun

**Affiliations:** † Department of Medical Biology, Cerrahpasa Faculty of Medicine, Istanbul University-Cerrahpasa, Istanbul 34098, Turkey; ‡ Brain and Neurodegenerative Disorders Research Laboratories, Department of Neuroscience, Institute of Neurological Sciences, Istanbul University-Cerrahpasa, Istanbul 34098, Turkey

## Abstract

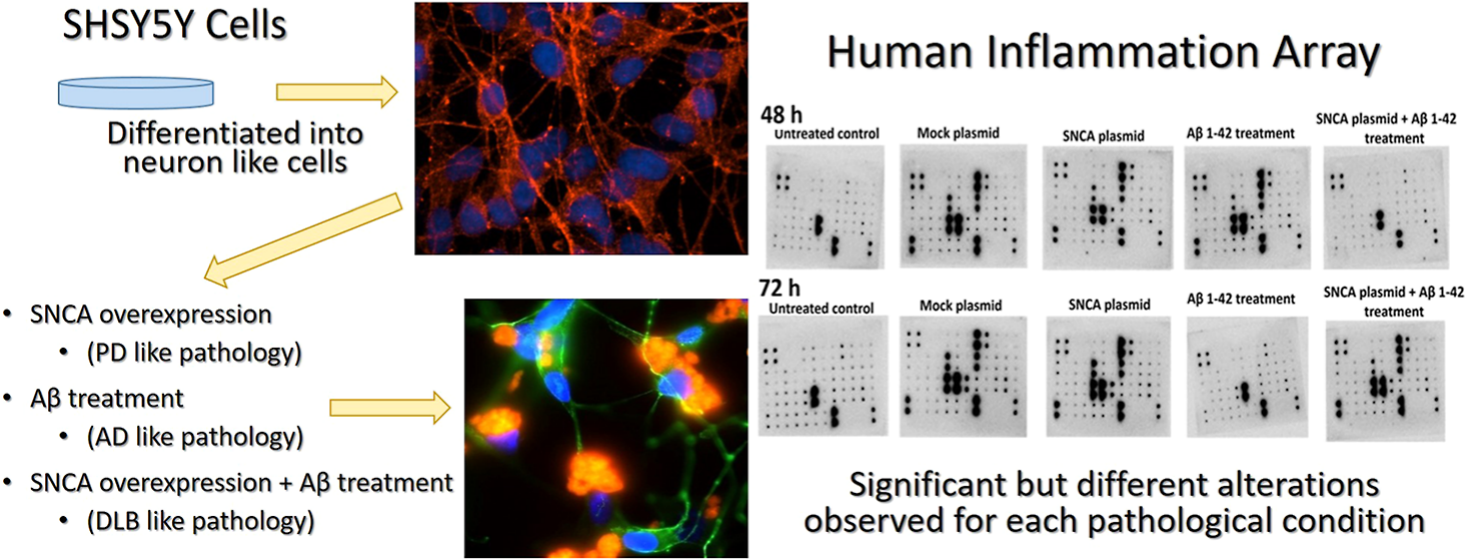

Neurodegeneration is a condition in which the neuronal structure and functions are altered with reduced neuronal survival and increased neuronal death in the central nervous system (CNS). Amyloid-β (Aβ) is the pathological hallmark of a common neurodegenerative disorder, Alzheimer disease. Parkinson disease and dementia with Lewy bodies are among α-synucleinopathies characterized by abnormal accumulation of insoluble α-synuclein protein. Neuroinflammation is seen in those neurodegenerative disorders regulated by cytokines and chemokines released from neurons, microglia, and astrocytes. Our study aimed to (1) define steady-state levels of cytokines and immune response modulators in SH-SY5Y cells that were differentiated into neuron-like cells and (2) compare the levels of target cytokines in cellular models of neurodegenerative disorders, namely, AD, PD, and DLB-like pathologies. AD, PD, and DLB-like pathologies were established by 6 μM Aβ1–42 administration, SNCA (α-synuclein) overexpression, and SNCA overexpression was followed by Aβ1–42 treatment, respectively. Alterations in the levels of 40 released inflammatory proteins (IPs) were analyzed by chemiluminescence-based Western/dot blot. Overexpression of human α-synuclein and administration of Aβ1–42 significantly changed the profile of IPs secretion, with particularly significant changes in CSF2, CCL5, CXCL8, CXCL10, ICAM1, IL1B, and IL16. Bioinformatics analysis revealed possible interactions between α-synuclein and IL1B. While TGF1, CCL2, TNF, IL10, IL4, and IL1B IPs were associated with Aβ 1–42, Aβ 1–42 treatment together with α-synuclein, overexpression is associated only with the IL6 protein. Consequently, AD, PD, and DLB-like pathologies might exert significant but different alterations in the inflammatory response.

## Introduction

Neurodegeneration is a common component of CNS disorders and affects the neuron function, structure, and survival. In neurodegenerative diseases, specific types of neurons and glial cells undergo degeneration, leading to the appearance of specific disease symptoms. AD and PD are among the most common neurodegenerative diseases.^1^ DLB is the most common type of dementia after AD.^2^

The most prominent pathological features of AD include amyloid plaques, the main component of the extracellular domain, and neurofibrillary tangles of accumulation of hyperphosphorylated tau protein in the intracellular domain.^3^ Soluble forms of Aβ cause neuronal dysfunction and play a significant role in stimulating pro-inflammatory activation of the primary microglia. Furthermore, compared to the fibrillar forms of Aβ, oligomer Aβ plaques induce the production of more or different pro-inflammatory cytokines in vitro microglia and astrocytes.^4^

α-Synucleinopathy describes neurodegenerative diseases characterized by abnormal accumulation of α-synuclein protein insoluble in neuronal or glial cells.^5,6^ These diseases include PD, DLB, and multisystem atrophy.^7^ α-Synuclein is a 14 kDa cytosolic protein located predominantly at the presynaptic terminals of neurons in the hippocampus, striatum, thalamus, cerebellum, and neocortex.^6^ The α-synuclein may form many conformations containing amyloidogenic oligomers.^8^ Mutation and an increase in the concentration of α-synuclein lead to misfolding of the protein. These misfolded α-synuclein aggregates stimulate glial cells and other immune-inflammatory cells, inducing neurodegeneration in the substantia nigra, releasing inflammatory cytokines and chemokines.^9^ PD is the second most common neurodegenerative disease characterized by the loss of dopaminergic neurons in the substantia nigra and the formation of α-synuclein-containing inclusions in neuronal soma and neurites called Lewy-associated α-synuclein pathologies (LRPs).^10,11^ Lewy body pathology is observed in approximately half of the patients with AD. Studies using transgenic mouse models have shown that Aβ enhances α-synuclein fibrillation both in vitro and in vivo.^12^ In another study with transgenic mice expressing both Aβ and α-synuclein and mice expressing α-synuclein alone, transgenic mice expressing both Aβ and α-synuclein had severe deficits in learning and memory and more Lewy body pathology in comparison to those expressing α-synuclein alone. These studies suggested a synergistic relationship between Aβ and α-synuclein.^13^ Further studies are required to examine whether α-synuclein interacts directly with Aβ, inhibits Aβ accumulation, increases the levels of toxic Aβ oligomers, and contributes to neuronal dysfunction.^12^ DLB is the second most common α-synucleinopathy after AD, although it is the most common type of dementia and shares many clinical features with AD.^10,14^ Therefore, it is likely that neuroinflammatory processes in AD are also involved in driving neurodegeneration in DLB.^15^ Although DLB patients have less loss of dopaminergic neurons in the substantia nigra, many patients have a significant accumulation of Aβ in the striatum, hippocampus, and cortex.^10^ Therefore, it is assumed that neuroinflammatory processes in AD may cause neurodegeneration in DLB.

The term “neuroinflammation” has emerged to differentiate inflammatory reactions in CNS from inflammation in other tissues.^16^ Neuroinflammation is a protective mechanism that repairs CNS and is regulated by neurons, microglia, astrocytes, other immune system cells, and various regulators released from these cells.^9^ Recovery of damaged tissue is enabled by low-grade acute inflammatory responses that remove agents, toxins, and dead cells. Although neuroinflammation is considered as a protective mechanism, extended inflammation contributes to tissue damage.^17^ Until recently, it was thought that neurons did not participate in the neuroinflammation process. However, there is evidence in the literature that neurons in neurodegenerative diseases such as AD contribute to the production of cytokines such as IL-1β, IL-6, and TNF, which may exacerbate local inflammation and neurodegeneration.^14,18,19^ Several studies have shown that inflammation and immune responses are the determining factors in disease progression and that familial and sporadic PD is responsible for pathogenic processes at the onset of the disease.^20^ Studies on PD suggest that high levels of IL-6 increase the risk of PD and may be associated with disease onset. These findings suggest that IL-6 may have a more complex relationship with disease progression, perhaps increasing the early and late stages of the disease.^21^

Although cytokines have traditionally been released from various cell types of the inflammatory system, they have recently been described as general signaling molecules. In addition to microglia, other glial cells, such as astrocytes and neurons, also release cytokines. Neurons are typically studied as a target for cytokine signaling pathways with various cytokine receptors expressed in nerve membranes.^22^ Cytokines and chemokines in the nervous system regulate neurodevelopment, neuroinflammation, and synaptic transmission.^23^ Neurons structurally express cytokines and their receptors in the CNS under normal and pathological conditions. Many studies have shown that long-term neuroinflammatory response is associated with the pathogenesis of neurodegenerative diseases such as AD, PD, and DLB, leading to dysfunction of neurons and neuronal death.^9^

In the present paper, we report a depth analysis of secretion of inflammatory proteins (IPs) and modification of the IP profile by Aβ1–42 administration, α synuclein overexpression, and α-synuclein overexpression followed by Aβ1–42 treatment in a human differentiated SH-SY5Y cell line.

## Materials and Methods

### SH-SY5Y Cell Culture Experiments

The SH-SY5Y cell line is the most frequently used cell line in AD and PD model studies. At the same time, as a result of applications induced by agents, such as retinoic acid and BDNF, they are similar to primary neurons.^24^ Human SH-SY5Y cells were obtained from ATCC CRL2266. SH-SY5Y cells were cultured in MEM (Gibco, 319502)/F12 (Gibco, 21765-029) (1:1, v/v), (10% fetal bovine serum (Gibco 10270-098)), 1% MEM Nonessential Amino Acids (Gibco, 11140-050), and 1% sodium pyruvate (Sigma P5280-256). Cells were subcultured when they reached 80–90% confluency. Cells were split into to 6 well plates 1 × 10^6^ cells/cm^2^ and incubated at 37 °C and 5% CO_2_ in a humidified atmosphere. When the cell confluency reached 40–50%, we added the differentiated cell culture medium into the 6 well plates and initiated the differentiation protocol. Differentiation medium consists of Neurobasal medium containing B27 supplement, NaCI, l-glutamine, putrescine, conalbumin, progesterone, insulin, 10 μM. All trans-retinoic acid (ATRA) and 50 ng/mL brain-derived neurotrophic factor (BDNF). On the fourth day of differentiation, the cell culture medium was replaced with fresh medium, and the differentiation protocol was terminated on the seventh day.

### Experimental Design

Following experimental groups were established: (1) untreated control group. This group was established to observe the baseline expression of target molecules in untreated differentiated SH-SY5Y cells. (2) Negative control or MOCK plasmid treated group, which was treated with mock plasmid containing CMV promoter but no open reading frame (ORF) for any gene (pMVC6-Entry, mammalian vector, oriGene cat no: PS100001); this group was used to distinguish between mimicry and inhibitory/activatory effects and true effect caused by α-synuclein transfection 3. SNCA overexpression group consisted of the cells that were transfected with *SNCA* gene (human synuclein, α (non A4 component of amyloid precursor), transcript variant 1; SNCA NM_000345 Human Tagged ORF Clone TrueORFGold oriGene cat no: RC210606). This group represented one of the pathological conditions of Parkinson disease. 4. Aβ-treated group was the cells that were treated with 6 μM Aβ1–42, representing the Alzheimer disease like pathology. 5. α-Synuclein overexpression followed by Aβ treated SH-SY5Y cell group: human SH-SY5Y cell culture in which DLB-like disease model was formed by administration of 6 μM Aβ1–42 24 h after the overexpression of human SNCA plasmid. All transfections and Aβ1–42 peptide treatment were performed on the 7 th day of differentiation (when cell confluency is 70%). All cell culture studies were performed three times independently on different days.

### α-Synuclein (SNCA) Overexpression

A stable cationic polymer polyethyleneimine (PEI), was prepared by diluting plasmids in NBM at 1:250 (μg/μL) and adding 3:1 ratio of PEI to DNA (w/w) according to the manufacturer’s protocol. Cells were transfected with the SNCA plasmid or mock plasmid for 48 and 72 h and protein isolation was performed at 48 or 72 h of treatments and the supernatants were collected at the same time points. Transient transfection of differentiated SH-SY5Y cell was performed and transfection success was monitored at 72 h of treatments with Western blot or immunofluorescence labeling.

### Aβ Administration

Aβ1–42 (Millipore AG912) was prepared as previously described^25−27^ and administered to culture media as 6 μM. This preparation was demonstrated to contain Aβ fibrils along with protofibrils and SDS-stable oligomers.^26^ Corresponding volume of PBS, the solvent of Aβ, was applied to the culture to establish vehicle groups. The large fibrillary aggregates formed by Aβ1–42 on the cells and neurites were visualized by fluorescence microscopy with Congo red staining as previously described.^25,27^

### Confirmation of SNCA Overexpression by Western Blotting

Total proteins were isolated with M-PER Mammalian Protein extraction reagent (Thermo Scientific 78501) as previously described.^25,28^ Thirty microgram of protein per sample was separated by electrophoresis and transferred to a PVDF membrane. Membranes were blocked with 5% dry milk overnight at +4 °C and then incubated with primary antibody overnight at +4 °C followed by the secondary antibody for 2 h at room temperature. Mouse polyclonal antibody to SNCA (Thermo Scientific, Thermo Fisher Scientific Inc. Illinois, 32-8100) at 1:250 was used as the primary antibody. Corresponding HRP conjugated secondary antibody was used at a dilution of 1:3000. Rabbit polyclonal to β actin (Abcam Ab8227) was used as a loading control for each PVDF membrane at a dilution of 1:3000. The signals were detected with the Lumi-LightPLUS Western Blotting Substrate (Roche 12015196 001). The target protein and actin β as a loading control were assessed on the same membranes. Protein band intensities were analyzed with ImageJ software for quantification. Protein band intensities were analyzed with ImageJ software for quantification. Western blot experiments were carried out by three independent researchers.

### Cellular Characterization and the Confirmation of *SNCA* Overexpression by Immunofluorescence Labeling

Immunofluorescence labeling (IF) was used for the detection of α-synuclein as previously described. SH-SY5Y cells were fixed with 3.7% paraformaldehyde and then blocked with 30% goat serum in 0.01% T-PBS for 1 h at room temperature. Cells were incubated with primary antibody overnight at 4 °C and further processed with corresponding secondary antibody that was labeled with Alexa Fluor 488 (A11034, Thermo Fisher) in the dark for 1 h at room temperature. Primary antibody was: mouse monoclonal antibody to α-syn-211 (1:250, Thermo Scientific, Thermo Fisher Scientific Inc. Illinois, USA, 32-8100). Cellular characterization was also determined with Tyrosine hydroxylase (TH) and MAP2 (microtubule-associated protein 2) immunofluorescent labeling (IF). The antibodies were: monoclonal antibody to TH (Thermo Fisher Scientific, 701949) at a ratio of 1:75 and MAP2 (Invitrogen, MA5-12826) at a ratio of 1:500. The specificity of the primary antibodies mouse monoclonal antibody to α-syn-211, TH (TH), and MAP2 that we used in our experiments were verified with knockdown or relative expression as stated in the manufacturer’s Web sites.

Overlay images were obtained using a Lionheart FX Automatic Fluorescent Microscope with Gen5 software (BioTek, Winooski, USA). ImageJ 1.44a software was used to analyze immunofluorescent intensities. Briefly, at least ten images of random areas from each group were analyzed for α-synuclein expression; the corrected total cell fluorescence (CTCF) was determined and calculated as previously described.^29,30^ Immunofluorescence experiments were performed by two independent researchers.

### Cytotoxicity Assay

The amount of lactate dehydrogenase (LDH) released into the culture medium after 48 and 72 h of treatment, which represents the level of the cytotoxicity in all groups, was determined with Cytotoxicity Detection kit (Roche 11644793001) by ELISA as previously described.^30−32^ Each sample was measured in triplicate.

### Human Inflammation Antibody Array

The following parameters that were released into the culture medium were investigated by the chemiluminescence-based Western/dot blot method using human inflammation antibody array (Ray Biotech C-Series Human Inflammation Antibody Array C3 (cat. no. AAH-INF-3–8) according to the manufacturer’s protocol similar to the method performed in our previous study.^33^ The target molecules were: Eotaxin-1/CCL11, Eotaxin-2/MPIF-2/CCL24 (C–C motif chemokine 24), GCSF (granulocyte colony stimulating factor), GM-CSF (granulocyte macrophage colony stimulating factor), ICAM-1/CD54 (intercellular adhesion molecule 1), IFN-gamma (interferon gamma), I-309/TCA-3/CCL1 (C–C motif chemokine 1), IL-1 α (interleukin-1 α), IL-1 β (interleukin-1 β), IL-2 (interleukin-2), IL-3 (interleukin-3), IL-4 (interleukin-4), IL-6 (interleukin-6), IL-6R (interleukin-6 receptor subunit α), IL-7 (interleukin-7), IL-8/CXCL8 (interleukin-8), IL-10 (interleukin-10), IL-11 (interleukin-11), IL-12 p40 (interleukin-12 subunit β), IL-12 p70 (interleukin-12 subunit α), IL-13 (interleukin-13), IL-15 (interleukin-15), IL-16 (interleukin-16),, IL-17A (interleukin-17A), IP-10/CXCL10 (C-X-C motif chemokine 10), MCP-1 (C–C motif chemokine 2), MCP-2/CCL8 (C–C motif chemokine 8), M-CSF (Macrophage colony-stimulating factor 1), MIG/CXCL9 (C-X-C motif chemokine 9), MIP-1alfa/CCL3 (C–C motif chemokine 3), MIP-1β/CCL4 (C–C motif chemokine 4), MIP-1 delta/CCL15 (C–C motif chemokine 15), RANTES/CCL5 (C–C motif chemokine 5), TGF β 1 (transforming growth factor β-1 proprotein), TNF alfa (tumor necrosis factor), TNF β (lymphotoxin-α), TNFRI/TNFRSF1A (tumor necrosis factor receptor superfamily member 1A), TNFRII/TNFRSF1B (tumor necrosis factor receptor superfamily member 1B), PDGF-BB (platelet-derived growth factor subunit B), and TIMP-2 (metalloproteinase inhibitor 2). Experiments for protein arrays have three independent experiments each having two protein samples.

### Statistical Analysis

Initial analysis of growth factor expression and release was performed with RayBio Analysis Tool-AAH-INF-3–8. IP levels, Western blot band intensities, cytotoxicity assays, and CTCF values were compared using GraphPad InStat DTCG 3.06 (GraphPad Software, Inc. San Diego USA) or SPSS 24.0 software. Comparisons were performed according to whether the data are normally distributed and whether the difference between the SDs obtained is significant, first by one-way ANOVA, followed by Tukey Kramer Multiple Comparison tests for multiple comparisons, or first with Kruskal–Wallis and then with Dunn’s Multiple Comparison tests for multiple comparisons. Normality of the data were checked with Kolmogorov and Smirnov tests and the difference between SDs were checked with Bartlett’s test. Bonferroni adjustment was performed when required. *p* < 0.05 was accepted as statistically significant difference.

The CTCF values of cells labeled with anti-α-synuclein antibody were also used to determine an increase in α-synuclein expression increased. The upper value of the 95% CI of the CTCF values of the anti-α-synuclein labeling of the MOCK group was considered the threshold. The percentage of cells in the SNCA group with CTCF above the threshold of the MOCK group is 62%, indicating that the level of α-synuclein expression increased by 62% after transfection.

Since no estimation of the outcomes was present and the study is the first of its kind in the literature, it was not possible to estimate means of each target and thus not possible to perform a priori sample size calculation.

### Protein–Protein Interaction and Pathway Analysis

The protein–protein interaction and pathway analysis in this study were performed as previously described.^33^ Briefly, the target proteins of groups were analyzed to determine the most significant pathways by Reactome Pathway Browser 3.6.^34,35^ The altered cytokines or their receptors in treatments were used for the pathway analysis in STRING (https://string-db.org/).^36^

## Results

### Morphological and Microscopic Evaluation Confirmed the Differentiation in SH-SY5Y Cells

**Figure 1:**
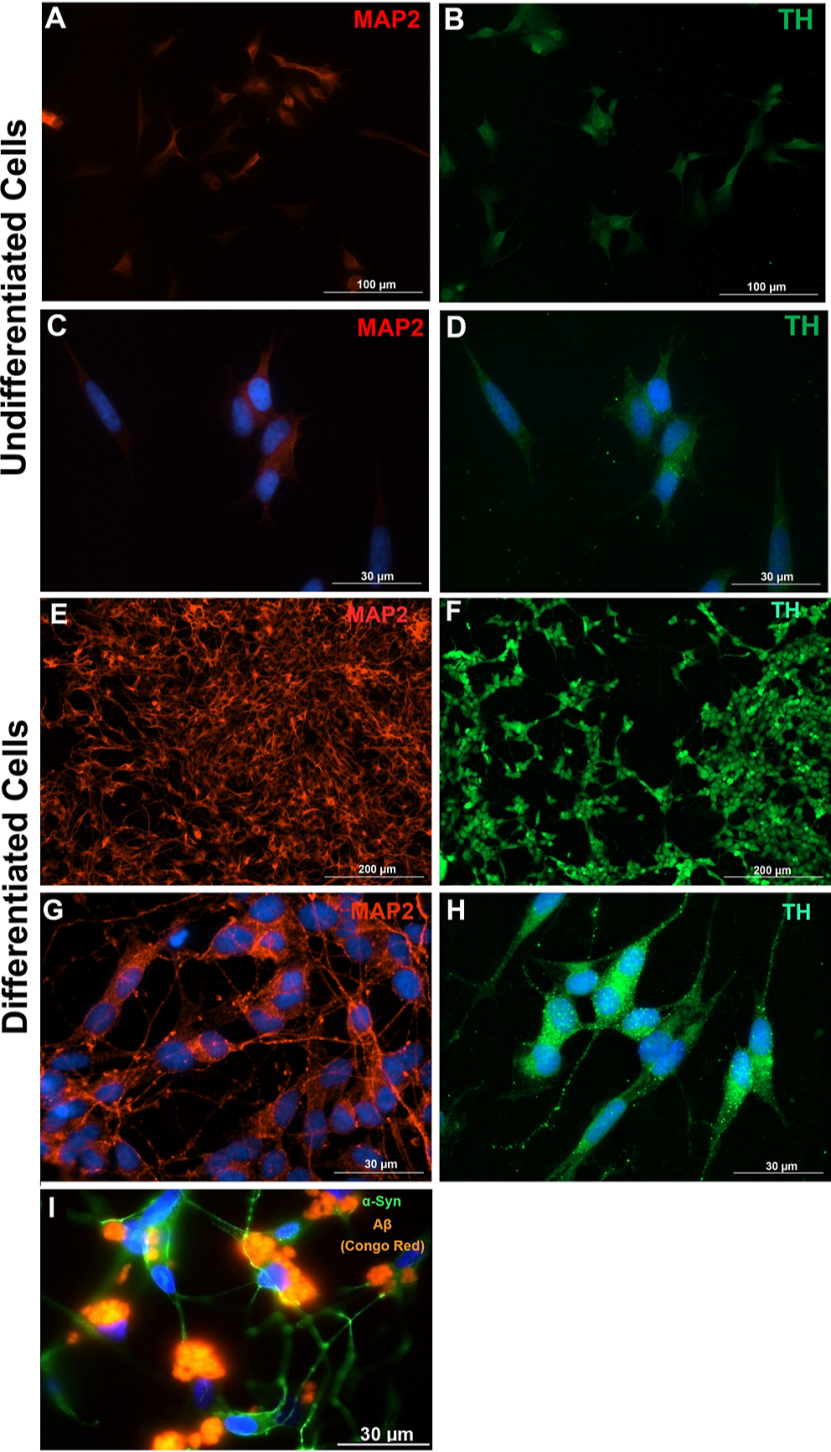
Morphological and microscopic evaluation confirmed the differentiation in SH-SY5Y cell and the aggregation of Aβ1–42 (A–I). TH and MAP2 immunofluorescence labeling indicated that the differentiated SH-SY5Y cells preserved their dopaminergic character and presented neuron like morphological properties with well-developed neurite. MAP2 and TH immunofluorescence labeling of undifferentiated cells (A,D). (A) MAP2 immunolabeling low magnification (20×), (B) TH immunolabeling low magnification (20×), (C) MAP2 immunolabeling high magnification (60×), and (D) TH immunolabeling high magnification (60×). MAP2 and TH immunofluorescence labeling of differentiated cells (E–H). (E) MAP2 immunolabeling at low magnification (10×), (F) TH immunolabeling at low magnification (10×), (G) MAP2 immunolabeling at high magnification (60×), (H) TH immunolabeling at high magnification (60×), TH (green), MAP2 (red), and DAPI (blue), a nuclear dye (I) demonstration of Aβ plaques and α-synuclein expression in human SH-SY5Y cells. Immunofluorescence labeling of α-synuclein and Congo red staining of amyloid β 1–42 indicated fibrillary aggregation of Aβ and α-synuclein expression in neuron-differentiated human SH-SY5Y cells. α-Synuclein (green), amyloid β 1–42 (orange), and nucleus (DAPI-blue). Magnification 100×.

Morphological evaluation was performed to observe whether differentiation was achieved in our experiments. The dopaminergic character was assessed with TH, and the neuronal character was assessed by microtubule associated protein 2 (MAP2) immunofluorescence labeling. Immunofluorescence labeling indicated low TH and MAP2 expression in undifferentiated SH-SY5Y cells (Figure 1A–D). However, the differentiated SH-SY5Y cells preserved their dopaminergic character and presented neuron-like morphological properties with well-developed neurites with relatively higher levels of TH and MAP2 expression (Figure 1E,H).

### Aβ Aggregations Demonstrated with Congo Red Staining

The presence of α-synuclein and the aggregation of Aβ 1–42 were demonstrated with α-syn immunofluorescence labeling and Congo red staining. Since Congo red binds to amyloids, aggregated as β-plated sheets, it is used for demonstration of the Aβ aggregations. Immunofluorescence labeling of α-synuclein and Congo red staining of amyloid β 1–42 indicated fibrillary aggregations of Aβ and the presence of α-synuclein expression in neuron differentiated human SH-SY5Y (Figure 1I).

### SNCA (α-Synuclein) Overexpression was Confirmed in Human Differentiated SH-SY5Y Cells

**Figure 2:**
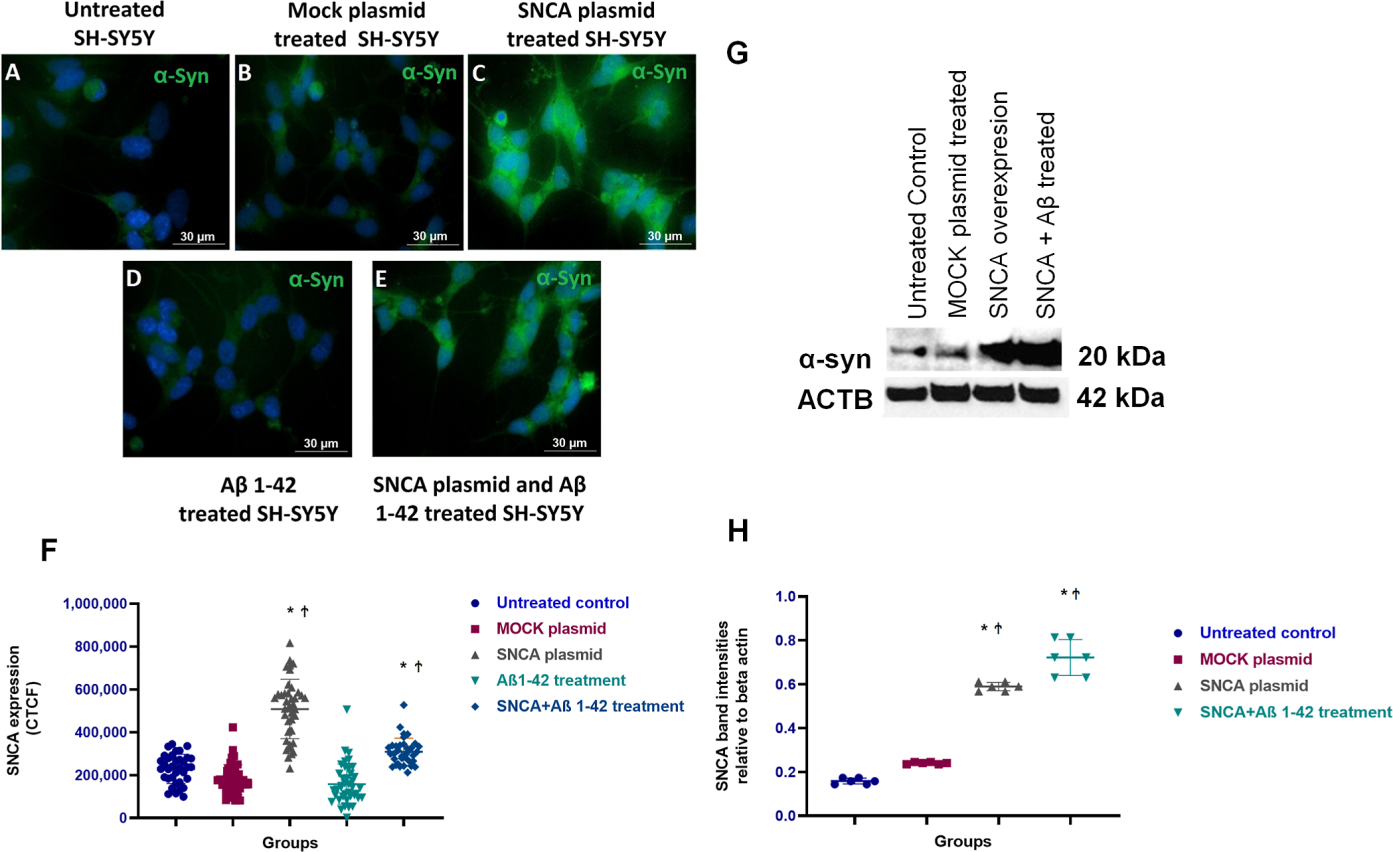
SNCA overexpression was confirmed with immunofluorescent and Western blot analysis. Expression of α-synuclein at 72 h in control, MOCK, SNCA, Aβ 1–42, and SNCA + Aβ 1–42 experimental groups (A–E). SNCA overexpression was demonstrated by SNCA antibody (green); nuclei were labeled with DAPI (blue). (A) Untreated control group, (B) MOCK group, (C) SNCA group, (D) Aβ 1–42 group, (E) SNCA + Aβ 1–42 group. (F) Evaluation of CTCF of α-synuclein between groups. The CTCF of α-synuclein increased significantly in the SNCA plasmid treated or SNCA + Aβ 1–42 group compared with the untreated control (*p < 0.001) or MOCK plasmid treated groups (^†^*p* < 0.05). Detailed statistical explanation given in Supporting Information. (G) Protein expression of target gene SNCA. (H) Comparison of SNCA protein band intensities relative to β actin. * significantly higher than that of control groups (*p* < 0.05). ^†^significantly higher than that of MOCK groups (*p* < 0.05). The data were gathered after 48 and 72 h of all treatment; detailed statistical explanation given in Supporting Information 1.

Expression of α-synuclein was determined in order to determine whether or not overexpression of SNCA was achieved in corresponding experimental groups. Transfection of cultured human SH-SY5Y cells with a plasmid containing the SNCA gene was successfully demonstrated by using immunofluorescence labeling as well as Western blotting with the α-syn antibody. Based on the results of immunofluorescence labeling and Western blotting, plasmid transfection was found to be successful at 72 h (Figure 2). Detailed statistical explanation is given in Supporting Information 1A.

Overexpression of α-synuclein was demonstrated by specific immunofluorescent labeling (Figure 2A–E). The CTCF of anti-α-synuclein labeling increased significantly in the SNCA group compared with both the untreated control and MOCK groups (Figure 2F). Overexpression was also confirmed by Western blotting (Figure 2H). The increase in α-synuclein expression, as determined by Western blotting, was as follows: untreated control group, 100%; MOCK plasmid-treated group, 152%; SNCA plasmid-treated group, 372%; and SNCA plasmid + Aβ1–42 treated group, 456%. The relative band intensities of α-synuclein in the SNCA-transfected groups were higher than in the untreated control and MOCK plasmid-treated groups. Detailed statistical explanation is given in Supporting Information 1A.

### Cytotoxicity Indicated Toxic Exposure for Amyloid β but Not for SNCA Overexpression

**Figure 3:**
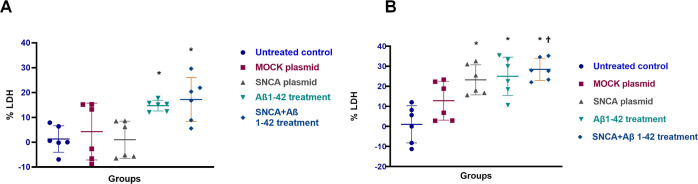
Cytotoxicity indicated toxic exposure for amyloid β but not for SNCA overexpression. LDH release of the untreated control group was accepted as 0%. (A) LDH levels of all groups at 48th h. LDH release of Aβ or SNCA plasmid + Aβ treated groups were significantly increased compared to control group (*p < 0.05, *p* < 0.05; respectively). (B) LDH levels of all groups at 72nd h. LDH release of SNCA, Aβ, or SNCA plasmid + Aβ treated groups were significantly increased compared to control group (*p < 0.01, *p* < 0.001, *p* < 0.001; respectively). LDH release in SNCA plasmid + Aβ treated groups was also increased compared to that in MOCK plasmid treated group (^†^*p* < 0.05).

Cytotoxicity measurements were performed in order to determine the level of damaged cells or cell death. The cytotoxic effect of α-synuclein overexpression and Aβ administration to human SH-SY5Y culture for 48 and 72 h was determined by measuring the amount of LDH released into the culture medium. Transfection with SNCA did not affect cell viability: no statistically significant difference was observed between LDH levels of the groups up to 48 and 72 h after transfection. Aβ administration or SNCA transfection, followed by Aβ administration, significantly increased LDH release. Changes in LDH release amounts of groups, mean LDH release values of groups, and statistical comparisons of differences between groups are shown in Figure 3.

### IP Profile Altered Significantly but Differently by Amyloid β,α-Synuclein or Amyloid β and α-Synuclein Combination

**Figure 4:**
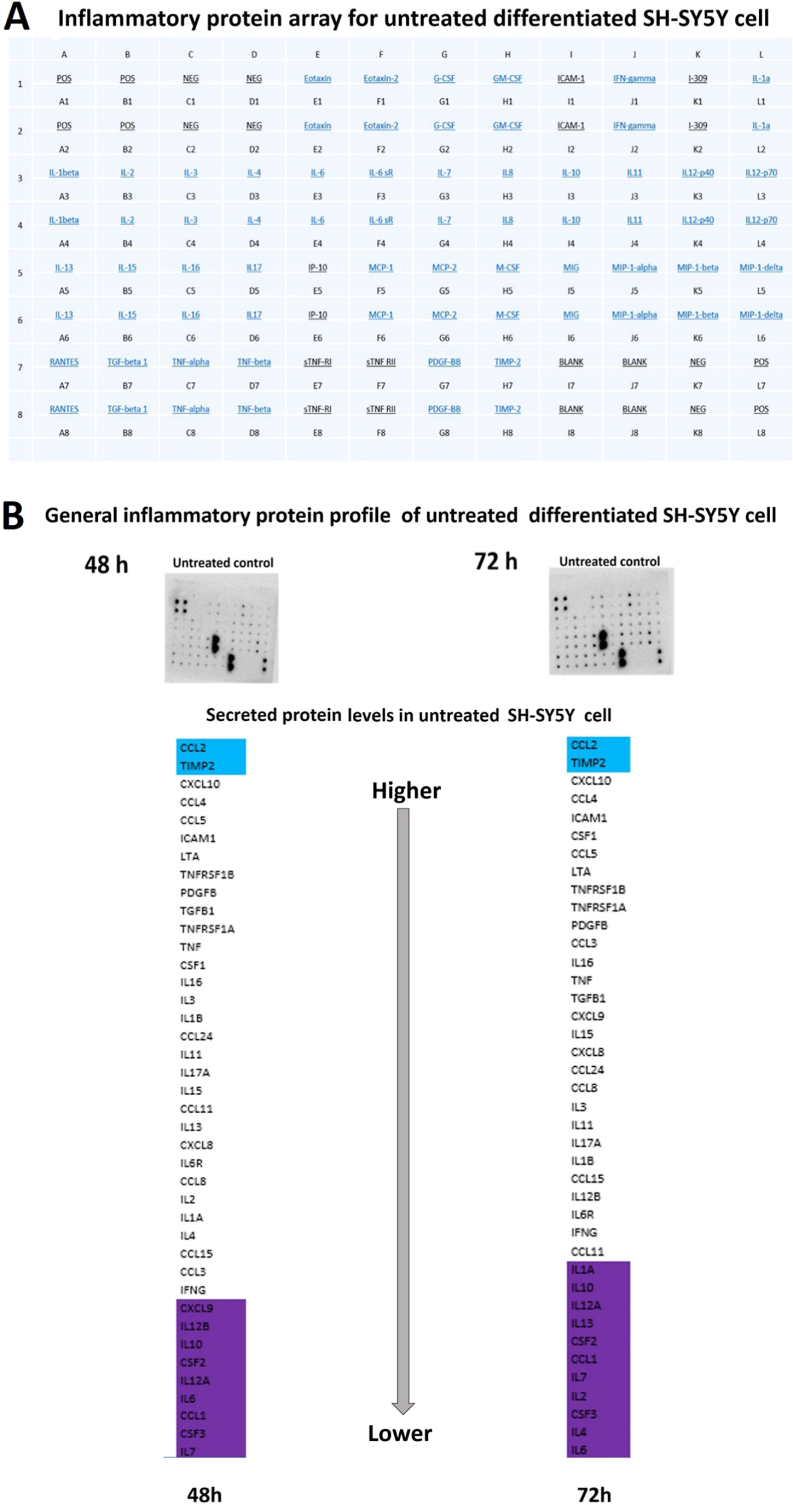
Steady state levels of cytokines indicate that CCL2 and TIMP2 are the highest released cytokines in untreated control groups. (A) The array and the dot blot membranes of untreated control, and the table with the target names and their positions were shown. (B) IP expression profile of untreated human SH-SY5Y cells. Blue color label proteins detected above the high threshold value, white color shows the proteins at the average values, and purple color shows the proteins below the low threshold value (95% CI) for secreted protein levels of untreated SH-SY5Y cell 48 h: (95% CI 0.1258–0.4592); 72 h (95% CI 0.2131–0.6674). Experiments for protein arrays has three independent experiments each having two protein samples. *N* = 6.

Ray Biotech C-Series Human Inflammation Antibody Array C-3 was used to determine the alterations in the inflammatory response profile of AD, PD, or DLB-like pathologies. Ray Biotech C-Series Human Inflammation Antibody Array C-3 analyses were performed in cell culture media samples obtained from all groups at 48 and 72 h. Using a dot blot array, we determined secreted levels of inflammatory factors in differentiated SH-SY5Y cell line. Results of our experiments are summarized in Table 1. The expression profile of IPs in the control SH-SY5Y cell line was refined by using 95% CI values of secreted IF levels of untreated human SH-SY5Y cell as cutoff values for upper or lower threshold levels. CCL2 and TIMP2 were secreted at higher levels by untreated human SH-SY5Y cells (the 95% CI for secreted protein levels of untreated SH-SY5Y cells at 48 h was 0.13–0.46, and at 72 h was 0.21–0.67) (Figure 4).

**Table 1:** Secreted Levels of Target Proteins 48 and 72 h of Treatments

target proteins (gene name-UniProtKB)	treatment periods (h)	control group band intensity-mean ± SD (*n* = 6)	mock group band intensity-mean ± SD (*n* = 6)	SNCA group band intensity-mean ± SD (*n* = 6)	Aβ group band intensity-mean ± SD (*n* = 6)	SNCA + Aβ group band intensity-mean ± SD (*n* = 6)	overall *p* value
CCL11 P51671	48	0.1675 ± 0.009047	0.1426 ± 0.009268	0.1886 ± 0.009369	0.1664 ± 0.01711	0.1337 ± 0.02479	<0.0001
	72	0.2142 ± 0.02582	0.1563 ± 0.02322	0.1808 ± 0.01893	0.1808 ± 0.01893	0.1288 ± 0.006127	<0.0001
CCL24 O00175	48	0.1745 ± 0.009562	0.182 ± 0.006332	0.229 ± 0.04111	0.1958 ± 0.0214	0.1705 ± 0.01383	0.0013
	72	0.2653 ± 0.03139	0.1536 ± 0.0231	0.2187 ± 0.02136	0.1538 ± 0.01101	0.1524 ± 0.01328	<0.0001
CSF3 P09919	48	0.07539 ± 0.007936	0.1623 ± 0.033	0.1972 ± 0.02691	0.1571 ± 0.02411	0.08168 ± 0.00597	<0.0001
	72	0.1061 ± 0.01094	0.2262 ± 0.03191	0.2684 ± 0.1086	0.07573 ± 0.01477	0.1379 ± 0.02016	<0.0001
CSF2 P04141	48	0.1022 ± 0.008521	2.571 ± 0.07356	3.295 ± 0.0564	2.934 ± 0.1268	0.09694 ± 0.01353	<0.0001
	72	0.1374 ± 0.01844	3.609 ± 0.1326	3.532 ± 0.1875	0.1647 ± 0.02075	2.889 ± 0.1018	<0.0001
ICAM1 P05362	48	0.2844 ± 0.01863	0.7813 ± 0.03843	1 ± 0.06118	0.9142 ± 0.03632	0.3568 ± 0.03234	<0.0001
	72	0.5509 ± 0.008343	1.31 ± 0.03465	1.117 ± 0.02324	0.5412 ± 0.01705	1.012 ± 0.03572	<0.0001
IFNG P01579	48	0.1275 ± 0.008074	0.1755 ± 0.004565	0.1573 ± 0.01516	0.1564 ± 0.01544	0.1278 ± 0.01724	<0.0001
	72	0.2263 ± 0.02678	0.2024 ± 0.009972	0.1416 ± 0.02372	0.1989 ± 0.01002	0.1658 ± 0.02377	<0.0001
CCL1 P22362	48	0.07636 ± 0.006841	0.1102 ± 0.005046	0.1177 ± 0.01398	0.1057 ± 0.006793	0.1007 ± 0.01023	<0.0001
	72	0.1349 ± 0.01861	0.1338 ± 0.03895	0.1339 ± 0.008284	0.1811 ± 0.01011	0.1335 ± 0.02927	0.0015
IL1A P01583	48	0.1416 ± 0.01802	0.1357 ± 0.01671	0.1881 ± 0.02724	0.1538 ± 0.01666	0.1324 ± 0.009292	<0.0001
	72	0.209 ± 0.02404	0.1935 ± 0.01439	0.1668 ± 0.02211	0.2145 ± 0.01012	0.1734 ± 0.04421	0.0017
IL1B P01584	48	0.197 ± 0.02895	0.3939 ± 0.05176	0.282 ± 0.007784	0.3729 ± 0.04185	0.1511 ± 0.02843	<0.0001
	72	0.2459 ± 0.02468	0.2832 ± 0.02977	0.4858 ± 0.008625	0.1937 ± 0.004566	0.4041 ± 0.003738	<0.0001
IL2 P60568	48	0.1433 ± 0.02469	0.1259 ± 0.04305	0.08449 ± 0.009307	0.164 ± 0.03647	0.1108 ± 0.0257	0.0003
	72	0.1177 ± 0.01991	0.08839 ± 0.01015	0.1084 ± 0.01312	0.121 ± 0.01122	0.1025 ± 0.01166	<0.0001
IL3 P08700	48	0.2034 ± 0.01437	0.1666 ± 0.01086	0.1342 ± 0.0117	0.2016 ± 0.01113	0.1519 ± 0.01275	<0.0001
	72	0.2519 ± 0.01326	0.1327 ± 0.006025	0.1714 ± 0.003812	0.1779 ± 0.02035	0.1747 ± 0.012	<0.0001
IL4 P05112	48	0.1361 ± 0.01406	0.08479 ± 0.00599	0.09891 ± 0.007907	0.1109 ± 0.0108	0.07063 ± 0.01208	<0.0001
	72	0.09035 ± 0.007725	0.08919 ± 0.00687	0.1114 ± 0.006427	0.07811 ± 0.006449	0.1056 ± 0.01357	<0.0001
IL6 P05231	48	0.09331 ± 0.01028	1.437 ± 0.03791	1.284 ± 0.03344	0.9207 ± 0.02927	0.05704 ± 0.002196	<0.0001
	72	0.08682 ± 0.007195	2.378 ± 0.1162	2.539 ± 0.05793	0.07309 ± 0.0213	1.139 ± 0.1118	<0.0001
IL6R P08887	48	0.1442 ± 0.0182	0.2441 ± 0.04217	0.2091 ± 0.01377	0.222 ± 0.05	0.1217 ± 0.008122	<0.0001
	72	0.2278 ± 0.04047	0.4004 ± 0.08547	0.4001 ± 0.05478	0.1558 ± 0.04013	0.2441 ± 0.0596	<0.0001
IL7 P13232	48	0.07417 ± 0.007993	0.08366 ± 0.005998	0.08726 ± 0.01144	0.1021 ± 0.012	0.06157 ± 0.004336	<0.0001
	72	0.1248 ± 0.008681	0.139 ± 0.01184	0.1538 ± 0.01803	0.07543 ± 0.01393	0.1134 ± 0.01829	<0.0001
CXCL8 P10145	48	0.1457 ± 0.01422	1.658 ± 0.07554	1.999 ± 0.1573	2.002 ± 0.1138	0.1431 ± 0.006966	<0.0001
	72	0.2681 ± 0.002746	2.598 ± 0.1169	2.507 ± 0.1316	0.3791 ± 0.01872	2.161 ± 0.08275	<0.0001
IL10 P22301	48	0.1184 ± 0.01786	0.1531 ± 0.02466	0.1191 ± 0.01514	0.1781 ± 0.03024	0.1012 ± 0.007659	<0.0001
	72	0.2002 ± 0.006299	0.2976 ± 0.03452	0.2129 ± 0.03233	0.2573 ± 0.03632	0.1941 ± 0.01822	<0.0001
IL11 P20809	48	0.1727 ± 0.02007	0.1909 ± 0.005762	0.2382 ± 0.007888	0.1992 ± 0.004728	0.1404 ± 0.01986	<0.0001
	72	0.2513 ± 0.007729	0.3284 ± 0.003779	0.3067 ± 0.005271	0.2359 ± 0.0116	0.1791 ± 0.01015	<0.0001
IL12B P29460	48	0.1185 ± 0.002569	0.1566 ± 0.01173	0.1638 ± 0.003389	0.1441 ± 0.002749	0.1215 ± 0.01149	<0.0001
	72	0.2375 ± 0.005424	0.2654 ± 0.01016	0.2546 ± 0.006827	0.1973 ± 0.01524	0.1287 ± 0.007681	<0.0001
IL12A P29459	48	0.09948 ± 0.01147	0.09213 ± 0.003577	0.1465 ± 0.00406	0.1356 ± 0.02757	0.1137 ± 0.005451	<0.0001
	72	0.1793 ± 0.004975	0.1688 ± 0.002426	0.17 ± 0.01491	0.1741 ± 0.009165	0.1101 ± 0.006949	<0.0001
IL13 P35225	48	0.1548 ± 0.02674	0.2368 ± 0.02974	0.1915 ± 0.02107	0.2088 ± 0.02736	0.0896 ± 0.005975	<0.0001
	72	0.1419 ± 0.01217	0.137 ± 0.03384	0.2062 ± 0.02781	0.1098 ± 0.008633	0.1653 ± 0.03412	<0.0001
IL15 P40933	48	0.1676 ± 0.02865	0.2171 ± 0.01197	0.1651 ± 0.003977	0.2233 ± 0.0183	0.1378 ± 0.002705	<0.0001
	72	0.2844 ± 0.009392	0.1374 ± 0.009346	0.1773 ± 0.009184	0.1792 ± 0.01074	0.1524 ± 0.008305	<0.0001
IL16 Q14005	48	0.218 ± 0.01566	0.2167 ± 0.01334	0.1856 ± 0.00994	0.226 ± 0.01817	0.1711 ± 0.005482	<0.0001
	72	0.3377 ± 0.01366	0.1561 ± 0.003385	0.2229 ± 0.002064	0.1938 ± 0.00558	0.1983 ± 0.004206	<0.0001
IL17A Q16552	48	0.1701 ± 0.02658	0.2243 ± 0.01497	0.2147 ± 0.04071	0.2463 ± 0.01176	0.1186 ± 0.01144	<0.0001
	72	0.2461 ± 0.005455	0.1777 ± 0.03456	0.1891 ± 0.007378	0.1438 ± 0.01241	0.1688 ± 0.02592	<0.0001
CXCL10 P02778	48	0.3507 ± 0.01465	2.568 ± 0.0183	2.975 ± 0.0765	2.82 ± 0.05785	0.3262 ± 0.04088	<0.0001
	72	0.6228 ± 0.009696	3.6270 ± 07007	3.467 ± 0.1766	0.6623 ± 0.05007	3.005 ± 0.1508	<0.0001
CCL2 P13500	48	2.578 ± 0.1334	2.772 ± 0.08942	3.247 ± 0.08133	3.232 ± 0.1173	2.98 ± 0.09213	<0.0001
	72	3.671 ± 0.01833	3.903 ± 0.05908	3.616 ± 0.1937	3.222 ± 0.1689	3.149 ± 0.1516	<0.0001
CCL8 P80075	48	0.1436 ± 0.04386	0.462 ± 0.04338	0.6393 ± 0.08936	0.7129 ± 0.0402	0.1679 ± 0.02161	<0.0001
	72	0.2592 ± 0.03975	1.697 ± 0.03002	1.43 ± 0.05232	0.268 ± 0.04465	0.8177 ± 0.03156	<0.0001
CSF1 P09603	48	0.2195 ± 0.0337	0.3293 ± 0.02194	0.302 ± 0.01778	0.4022 ± 0.01566	0.1926 ± 0.01731	<0.0001
	72	0.5192 ± 0.05886	0.6477 ± 0.03314	0.5719 ± 0.05512	0.5051 ± 0.04636	0.4425 ± 0.001002	<0.0001
CXCL9 Q07325	48	0.123 ± 0.01614	0.1223 ± 0.02075	0.104 ± 0.001831	0.1336 ± 0.002262	0.1156 ± 0.01146	0.0041
	72	0.2929 ± 0.01971	0.2004 ± 0.01261	0.1628 ± 0.005698	0.2185 ± 0.01536	0.1204 ± 0.002731	<0.0001
CCL3 P10147	48	0.1319 ± 0.01039	0.236 ± 0.00176	0.2163 ± 0.0028	0.2831 ± 0.009523	0.1231 ± 0.006525	<0.0001
	72	0.3563 ± 0.01413	0.5121 ± 0.01037	0.3956 ± 0.01697	0.2371 ± 0.01345	0.2352 ± 0.003417	<0.0001
CCL4 P13236	48	0.3376 ± 0.007383	0.4338 ± 0.006819	0.5468 ± 0.004149	0.5508 ± 0.0233	0.384 ± 0.01674	<0.0001
	72	0.603 ± 0.01062	0.8205 ± 0.01135	0.6646 ± 0.004489	0.5497 ± 0.01743	0.5378 ± 0.01046	<0.0001
CCL15 Q16663	48	0.1361 ± 0.009576	0.1449 ± 0.007285	0.1647 ± 0.003851	0.1557 ± 0.02373	0.1521 ± 0.002093	0.0002
	72	0.2451 ± 0.03294	0.22 ± 0.02035	0.1846 ± 0.01842	0.2368 ± 0.008111	0.1281 ± 0.005203	<0.0001
CCL5 P13501	48	0.3166 ± 0.0303	1.861 ± 0.02152	1.691 ± 0.1567	1.82 ± 0.05016	0.3051 ± 0.06326	<0.0001
	72	0.4707 ± 0.02944	2.362 ± 0.02986	2.234 ± 0.1515	0.392 ± 0.004035	2.295 ± 0.07585	<0.0001
TGFB1 P01137	48	0.2301 ± 0.05623	0.257 ± 0.03038	0.1188 ± 0.00978	0.1986 ± 0.01429	0.1468 ± 0.04783	<0.0001
	72	0.301 ± 0.00772	0.1953 ± 0.03717	0.2019 ± 0.01345	0.144 ± 0.01056	0.2193 ± 0.02536	<0.0001
TNF P01375	48	0.2199 ± 0.009489	0.2131 ± 0.007389	0.143 ± 0.006923	0.2136 ± 0.01579	0.1412 ± 0.003346	<0.0001
	72	0.3352 ± 0.01534	0.2317 ± 0.03516	0.2455 ± 0.01751	0.1985 ± 0.005967	0.2189 ± 0.01489	<0.0001
LTA P01374	48	0.2443 ± 0.0084	0.2682 ± 0.02066	0.1912 ± 0.01159	0.2668 ± 0.01064	0.2218 ± 0.002914	<0.0001
	72	0.4378 ± 0.03138	0.297 ± 0.03681	0.2871 ± 0.01021	0.2632 ± 0.006236	0.2073 ± 0.01327	<0.0001
TNFRSF1A P19438	48	0.2271 ± 0.009691	0.3267 ± 0.05712	0.3267 ± 0.03509	0.4757 ± 0.08382	0.1865 ± 0.005766	<0.0001
	72	0.3916 ± 0.0219	0.4937 ± 0.03	0.5779 ± 0.1143	0.2689 ± 0.01641	0.4103 ± 0.05946	<0.0001
TNFRSF1B P20333	48	0.2424 ± 0.06617	0.3984 ± 0.1142	0.3107 ± 0.05492	0.4218 ± 0.1245	0.2041 ± 0.03972	<0.0001
	72	0.3943 ± 0.04723	0.48 ± 0.08477	0.5335 ± 0.1004	0.3194 ± 0.04922	0.3929 ± 0.06216	<0.0001
PDGFB P01127	48	0.2421 ± 0.01555	0.2085 ± 0.0118	0.1604 ± 0.01649	0.2427 ± 0.05073	0.1958 ± 0.01061	<0.0001
	72	0.3825 ± 0.03281	0.3131 ± 0.03449	0.2934 ± 0.06426	0.3905 ± 0.02016	0.1669 ± 0.01889	<0.0001
TIMP2 P16035	48	2.452 ± 0.00797	2.428 ± 0.05889	2.507 ± 0.06864	2.675 ± 0.05724	2.343 ± 0.07165	<0.0001
	72	3.191 ± 0.08685	3.607 ± 0.05606	3.092 ± 0.09693	2.893 ± 0.06546	2.889 ± 0.1596	<0.0001

target proteins (gene name-UniProtKB)	treatment periods (h)	multiple vs test (MCT) *p* values of control (C) vs Aβ group (Aβ), mock (M) vs SNCA (S), SNCA (S) vs SNCA + Aβ group (S + Aβ), Aβ group (Aβ) vs SNCA + Aβ group (S + Aβ), respectively	results of Aβ group (control vs Aβ)	results of SNCA group (mock vs SNCA)	results of SNCA + Aβ group (SNCA vs SNCA + Aβ)	results of SNCA + Aβ group (Aβ vs SNCA + Aβ)
CCL11 P51671	48	C vs Aβ *P* > 0.05, M vs S *P* < 0.001, S vs S + Aβ *P* < 0.001, Aβ vs SNCA + Aβ *P* > 0.05		↑	↓	
	72	C vs Aβ *P* < 0.001, M vs S *P* > 0.05, S vs S + Aβ *P* < 0.05, Aβ vs SNCA + Aβ *P* > 0.05	↓		↑	
CCL24 O00175	48	C vs Aβ *P* > 0.05, M vs S *P* > 0.05, S vs S + Aβ *P* < 0.01, Aβ vs SNCA + Aβ *P* > 0.05			↓	
	72	C vs Aβ *P* < 0.001, M vs S *P* < 0.05, S vs S + Aβ *P* < 0.05, Aβ vs SNCA + Aβ *P* > 0.05		↑	↓	
CSF3 P09919	48	C vs Aβ *P* < 0.01, M vs S *P* > 0.05, S vs S + Aβ *P* < 0.001, Aβ vs SNCA + Aβ *P* > 0.05	↑		↓	
	72	C vs Aβ *P* > 0.05, M vs S *P* > 0.05, S vs S + Aβ *P* > 0.05, Aβ vs SNCA + Aβ *P* > 0.05				
CSF2 P04141	48	C vs Aβ *P* < 0.01, M vs S *P* < 0.05, S vs S + Aβ *P* < 0.001, Aβ vs SNCA + Aβ *P* < 0.01	↑	↓	↓	↓
	72	C vs Aβ *P* > 0.05, M vs S *P* > 0.05, S vs S + Aβ *P* > 0.05, Aβ vs SNCA + Aβ *P* > 0.05				
ICAM1 P05362	48	C vs Aβ *P* < 0.001, M vs S *P* > 0.05, S vs S + Aβ *P* < 0.001, Aβ vs SNCA + Aβ *P* < 0.05	↑		↓	↓
	72	C vs Aβ *P* > 0.05, M vs S *P* > 0.05, S vs S + Aβ *P* > 0.05, Aβ vs SNCA + Aβ *P* > 0.05				
IFNG P01579	48	C vs Aβ *P* > 0.05, M vs S *P* > 0.05, S vs S + Aβ *P* > 0.05, Aβ vs SNCA + Aβ *P* > 0.05				
	72	C vs Aβ *P* > 0.05, M vs S *P* < 0.01, S vs S + Aβ *P* > 0.05, Aβ vs SNCA + Aβ *P* > 0.05				
CCL1 P22362	48	C vs Aβ *P* < 0.05, M vs S *P* > 0.05, S vs S + Aβ *P* > 0.05, Aβ vs SNCA + Aβ *P* > 0.05	↑			
	72	C vs Aβ *P* < 0.05, M vs S *P* > 0.05, S vs S + Aβ *P* > 0.05, Aβ vs SNCA + Aβ *P* < 0.05	↑			↓
IL1A P01583	48	C vs Aβ *P* > 0.05, M vs S *P* < 0.001, S vs S + Aβ *P* < 0.001, Aβ vs SNCA + Aβ *P* > 0.05		↓	↓	
	72	C vs Aβ *P* > 0.05, M vs S *P* > 0.05, S vs S + Aβ *P* > 0.05, Aβ vs SNCA + Aβ *P* > 0.05				
IL1B P01584	48	C vs Aβ *P* < 0.01, M vs S *P* > 0.05, S vs S + Aβ *P* > 0.05, Aβ vs SNCA + Aβ *P* < 0.001	↑			↓
	72	C vs Aβ *P* > 0.05, M vs S *P* < 0.05, S vs S + Aβ *P* > 0.05, Aβ vs SNCA + Aβ *P* < 0.001		↓		↓
IL2 P60568	48	C vs Aβ *P* > 0.05, M vs S *P* > 0.05, S vs S + Aβ *P* > 0.05, Aβ vs SNCA + Aβ *P* > 0.05				
	72	C vs Aβ *P* > 0.05, M vs S *P* < 0.05, S vs S + Aβ *P* > 0.05, Aβ vs SNCA + Aβ *P* < 0.05		↑		↓
IL3 P08700	48	C vs Aβ *P* > 0.05, M vs S *P* < 0.001, S vs S + Aβ *P* > 0.05, Aβ vs SNCA + Aβ *P* < 0.001		↓		↓
	72	C vs Aβ *P* > 0.05, M vs S *P* > 0.05, S vs S + Aβ *P* > 0.05, Aβ vs SNCA + Aβ *P* > 0.05		↓		
IL4 P05112	48	C vs Aβ *P* < 0.001, M vs S *P* > 0.05, S vs S + Aβ *P* < 0.001, Aβ vs SNCA + Aβ *P* < 0.001	↓		↓	↓
	72	C vs Aβ *P* < 0.05, M vs S *P* < 0.001, S vs S + Aβ *P* > 0.05, Aβ vs SNCA + Aβ *P* < 0.001	↓	↑		↑
IL6 P05231	48	C vs Aβ *P* > 0.05, M vs S *P* > 0.05, S vs S + Aβ *P* < 0.001, Aβ vs SNCA + Aβ *P* < 0.05			↓	↓
	72	C vs Aβ *P* > 0.05, M vs S *P* > 0.05, S vs S + Aβ *P* > 0.05, Aβ vs SNCA + Aβ *P* > 0.05				
IL6R P08887	48	C vs Aβ *P* < 0.05, M vs S *P* > 0.05, S vs S + Aβ *P* < 0.01, Aβ vs SNCA + Aβ *P* < 0.001	↑		↓	↓
	72	C vs Aβ *P* > 0.05, M vs S *P* > 0.05, S vs S + Aβ *P* < 0.001, Aβ vs SNCA + Aβ *P* < 0.05			↓	↑
IL7 P13232	48	C vs Aβ *P* < 0.01, M vs S *P* > 0.05, S vs S + Aβ *P* < 0.01, Aβ vs SNCA + Aβ *P* < 0.001	↑		↓	↓
	72	C vs Aβ *P* < 0.001, M vs S *P* > 0.05, S vs S + Aβ *P* < 0.001, Aβ vs SNCA + Aβ *P* < 0.001	↓		↓	↑
CXCL8 P10145	48	C vs Aβ *P* < 0.001, M vs S *P* > 0.05, S vs S + Aβ *P* < 0.001, Aβ vs SNCA + Aβ *P* < 0.001	↑		↓	↓
	72	C vs Aβ *P* > 0.05, M vs S *P* > 0.05, S vs S + Aβ *P* > 0.05, Aβ vs SNCA + Aβ *P* > 0.05				
IL10 P22301	48	C vs Aβ *P* < 0.05, M vs S *P* > 0.05, S vs S + Aβ *P* > 0.05, Aβ vs SNCA + Aβ *P* < 0.001	↓			↓
	72	C vs Aβ *P* > 0.05, M vs S *P* < 0.01, S vs S + Aβ *P* > 0.05, Aβ vs SNCA + Aβ *P* < 0.01		↓		↓
IL11 P20809	48	C vs Aβ *P* > 0.05, M vs S *P* < 0.05, S vs S + Aβ *P* < 0.001, Aβ vs SNCA + Aβ *P* < 0.01		↑	↓	↓
	72	C vs Aβ *P* > 0.05, M vs S *P* > 0.05, S vs S + Aβ *P* < 0.001, Aβ vs SNCA + Aβ *P* > 0.05			↓	
IL12B P29460	48	C vs Aβ *P* > 0.05, M vs S *P* > 0.05, S vs S + Aβ *P* < 0.001, Aβ vs SNCA + Aβ *P* > 0.05			↓	
	72	C vs Aβ *P* > 0.05, M vs S *P* > 0.05, S vs S + Aβ *P* < 0.001, Aβ vs SNCA + Aβ *P* > 0.05			↓	
IL12A P29459	48	C vs Aβ *P* < 0.05, M vs S *P* < 0.001, S vs S + Aβ *P* > 0.05, Aβ vs SNCA + Aβ *P* > 0.05	↓	↓		
	72	C vs Aβ *P* > 0.05, M vs S *P* > 0.05, S vs S + Aβ *P* < 0.05, Aβ vs SNCA + Aβ *P* < 0.01			↓	↓
IL13 P35225	48	C vs Aβ *P* > 0.05, M vs S *P* > 0.05, S vs S + Aβ *P* < 0.01, Aβ vs SNCA + Aβ *P* < 0.001			↓	↓
	72	C vs Aβ *P* > 0.05, M vs S *P* < 0.01, S vs S + Aβ *P* > 0.05, Aβ vs SNCA + Aβ *P* < 0.01		↓		↓
IL15 P40933	48	C vs Aβ *P* < 0.05, M vs S *P* > 0.05, S vs S + Aβ *P* > 0.05, Aβ vs SNCA + Aβ *P* < 0.001	↓			↓
	72	C vs Aβ *P* < 0.001, M vs S *P* < 0.001, S vs S + Aβ *P* < 0.001, Aβ vs SNCA + Aβ *P* < 0.001	↓	↓	↓	↓
IL16 Q14005	48	C vs Aβ *P* > 0.05, M vs S *P* > 0.05, S vs S + Aβ *P* > 0.05, Aβ vs SNCA + Aβ *P* < 0.001				↓
	72	C vs Aβ *P* < 0.001, M vs S *P* < 0.001, S vs S + Aβ *P* > 0.05, Aβ vs SNCA + Aβ *P* > 0.05	↓	↓		
IL17A Q16552	48	C vs Aβ *P* < 0.01, M vs S *P* > 0.05, S vs S + Aβ *P* < 0.01, Aβ vs SNCA + Aβ *P* < 0.001	↓		↓	↓
	72	C vs Aβ *P* < 0.001, M vs S *P* > 0.05, S vs S + Aβ *P* < 0.05, Aβ vs SNCA + Aβ *P* > 0.05	↓			
CXCL10 P02778	48	C vs Aβ *P* < 0.05, M vs S *P* > 0.05, S vs S + Aβ *P* < 0.001, Aβ vs SNCA + Aβ *P* < 0.01	↓	↓	↓	↓
	72	C vs Aβ *P* > 0.05, M vs S *P* > 0.05, S vs S + Aβ *P* > 0.05, Aβ vs SNCA + Aβ *P* > 0.05				
CCL2 P13500	48	C vs Aβ *P* < 0.001, M vs S *P* < 0.01, S vs S + Aβ *P* > 0.05, Aβ vs SNCA + Aβ *P* > 0.05	↓	↓		
	72	C vs Aβ *P* > 0.05, M vs S *P* > 0.05, S vs S + Aβ *P* < 0.05, Aβ vs SNCA + Aβ *P* > 0.05			↓	
CCL8 P80075	48	C vs Aβ *P* < 0.001, M vs S *P* > 0.05, S vs S + Aβ *P* < 0.001, Aβ vs SNCA + Aβ *P* < 0.01	↓	↓	↓	↓
	72	C vs Aβ *P* > 0.05, M vs S *P* < 0.001, S vs S + Aβ *P* < 0.001, Aβ vs SNCA + Aβ *P* < 0.001		↓	↓	↓
CSF1 P09603	48	C vs Aβ *P* < 0.001, M vs S *P* > 0.05, S vs S + Aβ *P* < 0.001, Aβ vs SNCA + Aβ *P* < 0.001	↓		↓	↓
	72	C vs Aβ *P* > 0.05, M vs S *P* > 0.05, S vs S + Aβ *P* < 0.01, Aβ vs SNCA + Aβ *P* > 0.05			↓	
CXCL9 Q07325	48	C vs Aβ *P* > 0.05, M vs S *P* > 0.05, S vs S + Aβ *P* > 0.05, Aβ vs SNCA + Aβ *P* > 0.05				
	72	C vs Aβ *P* > 0.05, M vs S *P* > 0.05, S vs S + Aβ *P* > 0.05, Aβ vs SNCA + Aβ *P* < 0.001				↓
CCL3 P10147	48	C vs Aβ *P* < 0.001, M vs S *P* > 0.05, S vs S + Aβ *P* > 0.05, Aβ vs SNCA + Aβ *P* < 0.001	↑			↓
	72	C vs Aβ *P* > 0.05, M vs S *P* > 0.05, S vs S + Aβ *P* < 0.01, Aβ vs SNCA + Aβ *P* > 0.05			↓	
CCL4 P13236	48	C vs Aβ *P* < 0.001, M vs S *P* > 0.05, S vs S + Aβ *P* > 0.01, Aβ vs SNCA + Aβ *P* < 0.01	↑		↓	↓
	72	C vs Aβ *P* > 0.05, M vs S *P* > 0.05, S vs S + Aβ *P* < 0.01, Aβ vs SNCA + Aβ *P* > 0.05			↓	
CCL15 Q16663	48	C vs Aβ *P* > 0.05, M vs S *P* < 0.05, S vs S + Aβ *P* > 0.05, Aβ vs SNCA + Aβ *P* > 0.05		↓		
	72	C vs Aβ *P* > 0.05, M vs S *P* > 0.05, S vs S + Aβ *P* > 0.05, Aβ vs SNCA + Aβ *P* < 0.001				↓
CCL5 P13501	48	C vs Aβ *P* < 0.01, M vs S *P* > 0.05, S vs S + Aβ *P* > 0.05, Aβ vs SNCA + Aβ *P* < 0.01	↑		↓	↓
	72	C vs Aβ *P* > 0.05, M vs S *P* > 0.05, S vs S + Aβ *P* > 0.05, Aβ vs SNCA + Aβ *P* < 0.001				↑
TGFB1 P01137	48	C vs Aβ *P* > 0.05, M vs S *P* < 0.001, S vs S + Aβ *P* > 0.05, Aβ vs SNCA + Aβ *P* > 0.05		↓		
	72	C vs Aβ *P* < 0.001, M vs S *P* > 0.05, S vs S + Aβ *P* < 0.05, Aβ vs SNCA + Aβ *P* < 0.01	↓			↑
TNF P01375	48	C vs Aβ *P* > 0.05, M vs S *P* < 0.05, S vs S + Aβ *P* > 0.05, Aβ vs SNCA + Aβ *P* < 0.01	↓	↓		↓
	72	C vs Aβ *P* < 0.001, M vs S *P* > 0.05, S vs S + Aβ *P* > 0.05, Aβ vs SNCA + Aβ *P* > 0.05	↓	↓		
LTA P01374	48	C vs Aβ *P* > 0.05, M vs S *P* < 0.001, S vs S + Aβ *P* > 0.05, Aβ vs SNCA + Aβ *P* < 0.01		↓	↓	
	72	C vs Aβ *P* < 0.001, M vs S *P* > 0.05, S vs S + Aβ *P* < 0.01, Aβ vs SNCA + Aβ *P* > 0.05	↓		↓	
TNFRSF1A P19438	48	C vs Aβ *P* < 0.001, M vs S *P* > 0.05, S vs S + Aβ *P* < 0.01, Aβ vs SNCA + Aβ *P* < 0.001	↑		↓	↓
	72	C vs Aβ *P* > 0.05, M vs S *P* > 0.05, S vs S + Aβ *P* > 0.05, Aβ vs SNCA + Aβ *P* > 0.05				
TNFRSF1B P20333	48	C vs Aβ *P* < 0.01, M vs S *P* > 0.05, S vs S + Aβ *P* > 0.05, Aβ vs SNCA + Aβ *P* < 0.001	↑			↓
	72	C vs Aβ *P* > 0.05, M vs S *P* > 0.05, S vs S + Aβ *P* < 0.01, Aβ vs SNCA + Aβ *P* > 0.05			↓	
PDGFB P01127	48	C vs Aβ *P* > 0.05, M vs S *P* < 0.05, S vs S + Aβ *P* > 0.05, Aβ vs SNCA + Aβ *P* > 0.05			↓	
	72	C vs Aβ *P* > 0.05, M vs S *P* > 0.05, S vs S + Aβ *P* > 0.05, Aβ vs SNCA + Aβ *P* < 0.001				↓
TIMP2 P16035	48	C vs Aβ *P* > 0.05, M vs S *P* > 0.05, S vs S + Aβ *P* < 0.05, Aβ vs SNCA + Aβ *P* < 0.001			↓	↓
	72	C vs Aβ *P* < 0.01, M vs S *P* < 0.05, S vs S + Aβ *P* > 0.05, Aβ vs SNCA + Aβ *P* > 0.05	↓	↓		

Modifications of IF profile by amyloid β treatment, α-synuclein overexpression, and α-synuclein overexpression and α-synuclein overexpression + amyloid β treatment are presented in Table 1. At 48 h after amyloid β treatment in Aβ 1–42 treated group, secreted levels of CSF3, CSF2, ICAM1, CCL1, IL1B, IL6R, IL7, CXCL8, IL10, IL12A, IL15, IL17A, CXCL10, CCL2, CCL8, CSF1, CCL3, CCL4, CCL5, TNFRSF1A, and TNFRSF1B increased, while only IL4 decreased when compared with untreated control group after multiple comparisons test (MCT). At 72 h in Aβ 1–42 treated group, secreted levels of CCL11, CCL24, IL4, IL7, IL15, IL16, IL17A, TGFB1, TNF, LTA, and TIMP2 decreased, while only CCL1 levels increased when compared with untreated control group after MCT.

At 48 h after transfection in the SNCA plasmid-treated group, the levels of CCL11, CSF2, IL1A, IL11, IL12A, CCL2, and CCL15 increased, while IL3, TGFB1, TNF, and LTA decreased when compared with those of the MOCK group after MCT. At 72 h in the SNCA group, the secreted levels of IL1B, IL2, IL4, IL13, IL15, and IL16 increased, while those of IL3, IL10, CCL8, and TIMP2 decreased when compared with those of the MOCK group after MCT.

**Figure 5:**
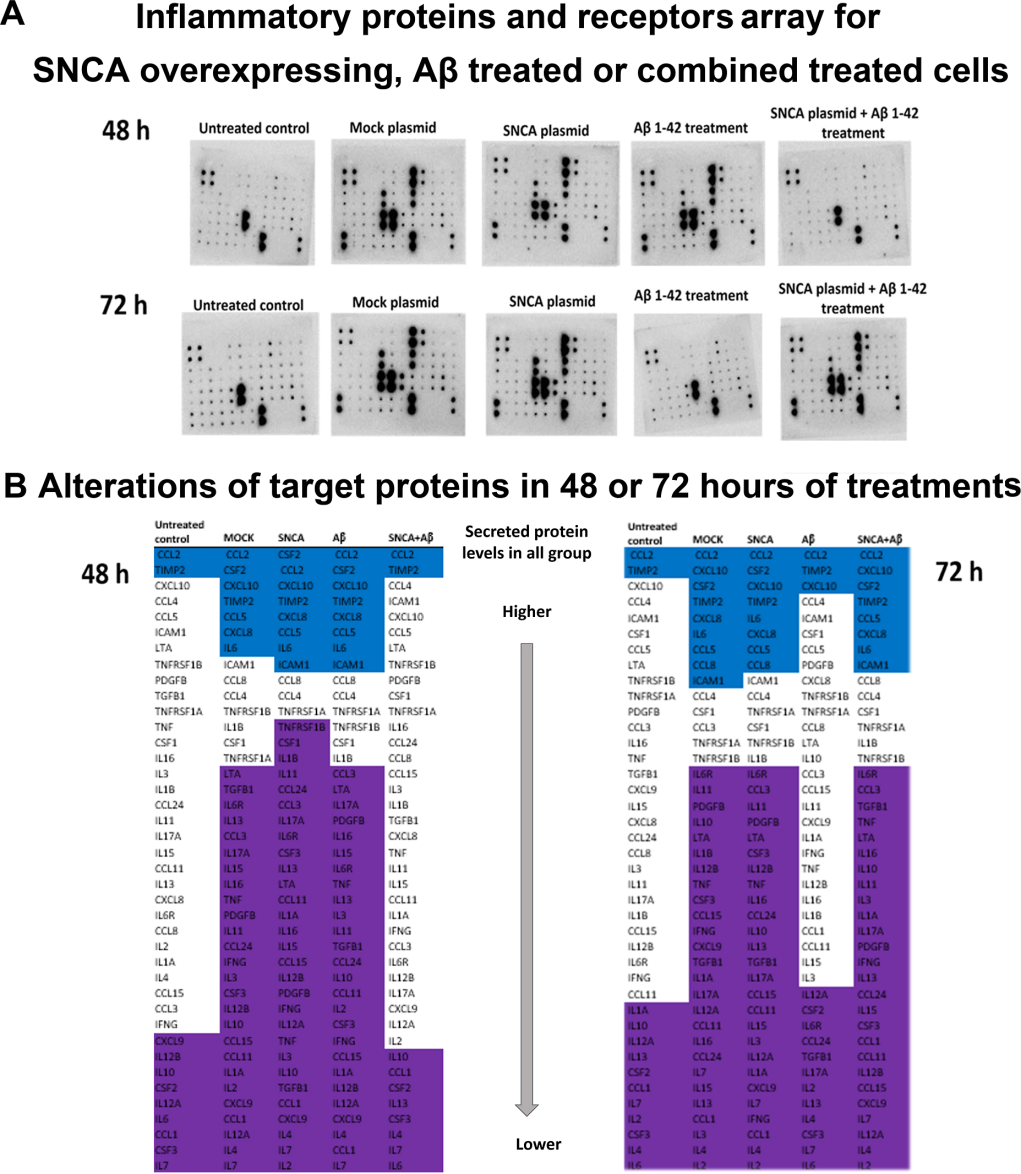
IP profile altered significantly but differently by amyloid β,α-synuclein, or amyloid β and α-synuclein combination. (A) The array and the dot blot membranes of untreated control, MOCK plasmid, SNCA plasmid SH-SY5Y cells, Aβ 1–42 treatment, and α-synuclein overexpression followed by Aβ 1–42 treatment were shown. (B) The alterations in the target proteins in 48 or 72 h of treatments. The 95% CI values of secreted IF protein levels in each group were set as the upper or lower threshold values for IF production. For the calculation of 95% CI, mean levels of IFs were used at 48 and 72 h. Blue indicates proteins detected above the high threshold value, white indicates the proteins at the average values, and purple indicates the proteins below the low threshold value. Experiments for protein arrays has three independent experiments each having two protein samples. *N* = 6.

At 48 h after SNCA plasmid and Aβ 1–42 treated group, secreted levels of CCL11, CCL24, CSF3, CSF2, ICAM1, IL1A, IL4, IL6, IL7, CXCL8, IL11, IL12B, IL17A, CXCL10, CCL8, CSF1, CCL4, CCL5, LTA, TNFRSF1A, PDGFB, and TIMP2 decreased when compared with the SNCA group. At 72 h in the SNCA plasmid and Aβ 1–42 treated group, secreted levels of CCL11 increased, whereas those of CCL24, IL6, IL7, IL11, IL12B, IL12A, IL15, CCL2, CCL8, CSF1, CCL3, CCL4, LTA, and TNFRSF1B decreased when compared with the SNCA group. At 48 h after SNCA plasmid and Aβ 1–42 treated group, secreted levels of CSF2, ICAM1, IL1B, IL3, IL4, IL6, IL6R, IL7, CXCL8, IL10, IL11, IL13, IL15, IL16, 1L17, CXCL10, CCL8, CSF1, CCL3, CCL4, CCL5, TNF, TNFRSF1A, TNFRSF1B, and TIMP2 decreased when compared with the Aβ 1–42 treated group. At 72 h after SNCA plasmid and Aβ 1–42 group secreted levels of IL1B, IL4, IL6R, IL7, IL13, CCL8, CCL5, and TGFB1 increased, while CCL1, IL2, IL10, IL12A, IL15, CXCL9, CCL15, and PDGFB decreased when compared with the Aβ 1–42 group. All the results are presented in Table 1 and summarized in Figure 5.

### Protein–Protein Interaction and Pathway Analysis Indicated Different Targets for α-Synuclein and Aβ 1–42

The 95% CI values of secreted IP levels were used to determine which IP is detected above the high threshold value. Accordingly in the control, MOCK, Aβ, and SNCA + Aβ groups CCL2, TIMP2, and CXCL10 were secreted at a level higher than the upper threshold. Addition to CCL2, TIMP2, CXCL10; CSF2, IL6, CXCL8, CCL5, and CCL8 were secreted at higher level than the upper threshold in SNCA group.

**Figure 6:**
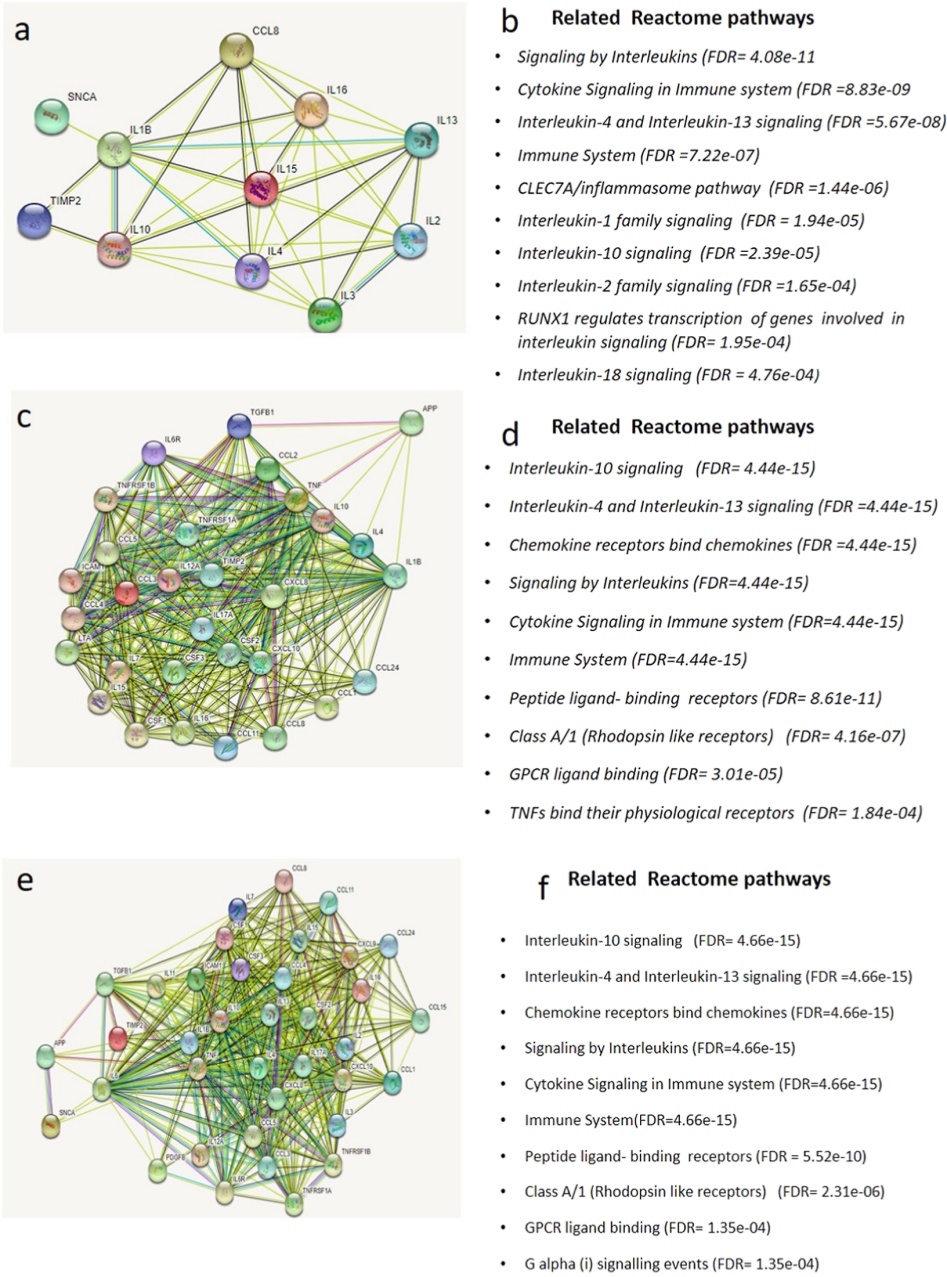
Protein–protein interaction and pathway analysis indicated different targets for α-synuclein and Aβ 1–42. (A,C,E) Secreted protein levels of which were changed Aβ 1–42 treatment, α-synuclein overexpressing, α-synuclein overexpressing, and Aβ 1–42 treatment SH-SY5Y cell. While STRING analysis shows that α synuclein is related only to IL1B, Aβ 1–42 treatment is related TGFB1, CCL2, TNF, IL10, IL4, and IL1B. In addition, α-synuclein overexpression and Aβ 1–42 treatment are associated with IL-6. (B,D,F) Top 10 related reactome pathways. Experiments for protein arrays have three independent experiments each having two protein samples. *N* = 6.

The levels of IPs in the extracellular medium in α-synuclein overexpressing, Aβ 1–42 treated, α-synuclein overexpressing, and Aβ 1–42 treated human SH-SY5Y cell are shown in Figure 5. We used the String for the pathway analysis. The analysis showed that there is almost no known information about interactions between synuclein and the inflammatory protein levels, of which change with the overexpression of α-synuclein, except predicted interaction with IL1B (Figure 6A). According to the analysis results, while TGF1, CCL2, TNF, IL10, IL4, and IL1B IPs were found to be associated with Aβ 1–42; it has been shown that Aβ 1–42 treatment together with α-synuclein overexpression is associated only with IL6 protein (Figure 6C,E respectively). Reactome analysis of these proteins showed significant pathways; the pathways and false discovery rate (FDR) scores are presented in Figure 6B,D,F and Supporting Information 1B.

## Discussion

This study reports the profiles of secreted IPs and their receptors in differentiated SH-SY5Y cells and is the first research to compare neurodegeneration and neurodegenerative diseases by creating conditions similar to the three most common neurodegenerative disease models: AD, PD, and DLB. However, a study comparing these three disease models in terms of IP profiles at the cellular level has not yet been reported. We analyzed the protein expression of 37 IPs and three IP receptors. We found secretion of IPs associated with signaling pathways such as signaling by interleukins, interleukin-4 and interleukin-13 signaling, cytokine signaling in immune system, and immune system according to Reactome pathway analysis tool. Secretion of IL6 was below the threshold, whereas CCL2 was the highest secreted chemokine under normal culture conditions.

We gathered the data for the effects of SNCA overexpression from the comparison between MOCK and SNCA (α synuclein overexpressing) groups to avoid false positive or negative results that may occur with the transfection of the plasmid backbone.^37^ All pathological conditions, including Aβ1–42 treatment, α synuclein overexpression, and α synuclein overexpression followed by Aβ1–42 treatment, affected the profile of IPs secretion levels. We detected that the secretion of most IPs increased in the Aβ group while IL4, CCL11, CCL24, IL4, IL7, IL16, TGFB1, TNF, LTA, and TIMP2 decreased. Also, the secretion of IL1B, IL2, IL4, IL13, IL15, and IL16 increased in the α synuclein overexpressing group, where IL3, IL10, CCL8, and TIMP2 decreased. However, in the SNCA + Aβ group, the secretion of most IPs decreased where CCL11, IL1B, IL4, IL6R, IL7, IL13, CCL8, CCL5, and TGFB1 increased.

According to our results, CCL2 is one of the highest secreted chemokines in SH-SY5Y cells. The SH-SY5Y cell line secretes significantly higher amounts of cytokines such as MCP-1 (CCL2), M-CSF (CSF1), TNF, and IL-12p40 (IL12A), IL-16.^38^ Our results revealed relatively high levels of CCL2 in all of the experimental groups. Basal release of chemokines such as CCL2 is required for the body to perform its normal physiological functions.^39^ CCL2 is a member of the CC chemokine family and can be expressed by astrocytes, microglia, and axotomized neurons. Initially, its role was suggested to promote macrophages’ infiltration into tumors. Instead, CCL2 is essential in regulating the CNS’s repair processes and cellular interactions.^40^ It also plays a role in Aβ degradation and clearance of neuronal damage.^41^ Multiple data obtained as a result of studies show that significant increases in MCP-1 predict AD, and this chemokine plays a critical role in the disease process.^40^ Various clinical data have demonstrated that MCP-1 levels increase in both CSF and serum of AD patients.^42^ MCP-1 is also closely associated with the pathogenesis of PD. A study revealed that the release of CCL2 levels increased when SH-SY5Y cells were incubated with α-synuclein to induce neuropathological lesions and the pathogenesis of PD.^43^ CCL2 is the most potent activator of CCR2 (C–C chemokine receptor type 2) signaling, and the upregulation of CCR2 by CCL2 is associated with inflammatory diseases in the central nervous system multiple sclerosis, Alzheimer disease, and ischemic stroke.^44,45^ Neurodegeneration and neuronal death in neurodegenerative diseases primarily cause increased proinflammatory regulators such as IL-1β, TNF, IL-6, IL-8, CCL2, and CCL5.^9^

We found high levels of CSF2 (GM-CSF) released by SH-SY5Y cells. Overexpression of α-synuclein increased the levels of CSF2. CSF2 affects neuronal plasticity, learning, and memory and has neurotrophic and antiapoptotic activities for photoreceptors. CSF2 plays a role in disease prevention in animal models of AD and is currently being approved in clinical trials because of its potential to reduce dementia. CSF2 protects neurons in MP models, including MPTP, 6-OHDA, and paraquat, by reducing neuronal apoptosis, Bcl-2, and Bax-related proteins and stimulating brain-derived neurotrophic factors.^46^

Members of the IL-1 family are expressed at low or undetectable levels in healthy brain. However, their expression is rapidly upregulated by various experimental brain injuries, including ischemia, trauma, hypoxia, and neurotoxic or inflammatory stimuli.^47^ It has been reported that IL-1β is upregulated in AD in some studies but down-regulated or unchanged in other studies.^48^ However, IL-1β levels in PD cerebrospinal fluids are significantly higher than those in individuals with other neurological diseases.^49^ In our previous study, we showed that serum IL-1β levels of patients with PD increased significantly compared to those in healthy individuals, late-onset Alzheimer disease, and MCI patients, while we have shown that it decreases in patients with early onset Alzheimer disease.^50^

The results of this study confer one type of cell and should be repeated in other cell lines or primary cultures.

## Conclusions

In summary, this is the first study to investigate the effect of amyloid-β and α-synuclein pathologies on the immune response in neuron-differentiated SH-SY5Y cells. Our data indicated that administration of α-synuclein and Aβ1–42 significantly changed the profile of IP secretion with particularly significant changes in CSF2, CCL5, CXCL8, CXCL10, ICAM1, IL1B, and IL16. We observed possible interactions between α-synuclein and IL1B. While TGF1, CCL2, TNF, IL10, IL4, and IL1B IPs were associated with Aβ 1–42. Aβ 1–42 treatment together with α-synuclein overexpression is associated only with IL6 protein. AD, PD, and DLB-like pathologies might exert significant but different alterations in inflammatory response.

## Data Availability

All data generated or analyzed during this study are included in this published article and its Supporting Information files.

## Supporting Information Available

Details of statistical analysis, and Reactome analysis; statistical analysis of SNCA overexpression confirmed by Western blot and immunofluorescence labeling; and top 10 Reactome pathway analysis results that may be associated with IPs released due to amyloid β, SNCA overexpression, SNCA overexpression, and amyloid β administration (PDF)
