# Supplementary material for: Amyloid β,α-Synuclein and Amyloid β-α-Synuclein Combination Exert Significant but Different Alterations in Inflammatory Response Profile in Differentiated Human SH-SY5Y Cells
Source: ACS Omega. 2023 Nov 22;8(48):45519–34. doi: 10.1021/acsomega.3c05585 (PMC10701882; doi:10.1021/acsomega.3c05585)
Supplement: Supplementary file 1 — ao3c05585_si_001.pdf [file ao3c05585_si_001.pdf]

Supporting Information for : Amyloid beta, alpha-synuclein and amyloid beta-alpha- synuclein combination exert significant but different alterations in inflammatory response profile in differentiated human SH-SY5Y cells.

Ebru Keskin [1], Duygu Gezen-Ak[2], Erdinç Dursun[2]

[1] Department of Medical Biology, Cerrahpasa Faculty of Medicine, Istanbul University-Cerrahpasa, Istanbul, Turkey

[2] Brain and Neurodegenerative Disorders Research Laboratories, Department of Neuroscience, Institute of Neurological Sciences, Istanbul University-Cerrahpasa, Istanbul, Turkey

Running title: Aβ or α-synuclein induced inflammatory response in human neuronal cells

***Correspondence to:** Erdinç Dursun

Address: Department of Neuroscience, Institute of Neurological Sciences,

Istanbul University-Cerrahpasa, 34098, Istanbul, Turkey.

Phone: +90 212 414 30 00/22032, +90 533 339 98 82

Fax: +90 212 414 30 42

Ebru Keskin, ksknebru@gmail.com

Duygu Gezen-Ak duygugezenak@iuc.edu.tr

Erdinç Dursun erdinc.dursun@iuc.edu.tr

Supplementary material 1A: Details of statistical analysis.

SNCA western analysis: Bartlett statistic (corrected) = 16.904, The P value is 0.0020. Bartlett's test suggests that the differences among the SDs is significant. Kolmogorov and Smirnov indicated all data distributed normal according to Gaussian distributions. Given that, first Kruskal Wallis and then with Dunn's Multiple Comparison tests were performed. Df: 3. N=6.

Group	Median	Min	Max
Control	1.505	1.049	2.120
Mock	1.981	1.401	2.741
SNCA	3.537	2.831	4.441
ABETA	2.037	2.032	2.042
SNCA+ ABETA	1.736	1.393	2.175

Table S1: Statistical analysis of the western blot.

SNCA immunofluorescence (CTCF) analysis: Bartlett statistic (corrected) = 26.026, The P value is < 0.0001. Bartlett's test suggests that the differences among the SDs is significant. Kolmogorov and Smirnov indicated all data distributed normal according to Gaussian distributions. Given that, first Kruskal Wallis and then with Dunn's Multiple Comparison tests were performed. Df: 4. N for SNCA CTCF analysis were untreated control 99 cells, mock 126 cells, snca 120 cells, amyloid beta 117 cells, snca+ amyloid beta 99 cells.

Group	Median	Min	Max
Control	236861	99035	336141
Mock	174004	81597	423163
SNCA	526428	231888	817587
ABETA	140310	2397.3	507388
SNCA+ ABETA	301064	208191	528250

Table S2: Statistical analysis of the immunofluorescence.

Supplemantary Material 1B: Reactome analysis results. Secreted levels of 40 IPs in α-synuclein overexpression, Aβ1-42 treated , α-synuclein overexpression and Aβ treated human SH-SY5Y cells were used for analysis. The top 10 related reactome pathway results of secreted proteins were given respectively.

The following table shows the 10 most relevant pathway results of α-synuclein overexpression sorted by p-value (Figure 6B).

https://reactome.org/PathwayBrowser/#/ANALYSIS=MjAyMjA3MjkxODQ0NTZfMjIwMDg%3D

Pathway name	Entities				Reactions	
	found	ratio	p-value	FDR*	found	ratio
Signaling by Interleukins	12 / 658	0.043	6.58e-13	4.08e-11	114 / 505	0.036
Cytokine Signaling in Immune system	12 / 1,107	0.073	2.85e-10	8.83e-09	114 / 725	0.052
Interleukin-4 and Interleukin-13 signaling	7 / 211	0.014	2.84e-09	5.67e-08	27 / 47	0.003
Immune System	14 / 2,698	0.178	4.81e-08	7.22e-07	119 / 1,644	0.118
CLEC7A/inflammasome pathway	3 / 8	5.28e-04	1.20e-07	1.44e-06	2 / 4	2.87e-04
Interleukin-1 family signaling	5 / 183	0.012	1.94e-06	1.94e-05	24 / 92	0.007
Interleukin-10 signaling	4 / 86	0.006	2.99e-06	2.39e-05	12 / 15	0.001
Interleukin-2 family signaling	3 / 47	0.003	2.36e-05	1.65e-04	33 / 59	0.004
RUNX1 regulates transcription of genes involved in interleukin signaling	2 / 7	4.62e-04	3.25e-05	1.95e-04	2 / 5	3.59e-04
Interleukin-18 signaling	2 / 11	7.26e-04	8.01e-05	4.76e-04	1 / 4	2.87e-04

Table S3: Demonstration of the top 10 relevant pathway results of α-synuclein overexpression.

The following table shows the 10 most relevant pathway results of Aβ 1-42 treatment sorted by p-value (Figure 6D).

https://reactome.org/PathwayBrowser/#/ANALYSIS=MjAyMjA3MjkxODU3NDNfMjIwMTI%3D

Pathway name	Entities				Reactions	
	found	ratio	p-value	FDR*	found	ratio
Interleukin-10 signaling	32 / 86	0.006	1.11e-16	4.44e-15	12 / 15	0.001
Interleukin-4 and Interleukin-13 signaling	26 / 211	0.014	1.11e-16	4.44e-15	20 / 47	0.003
Chemokine receptors bind chemokines	12 / 57	0.004	1.11e-16	4.44e-15	8 / 19	0.001
Signaling by Interleukins	48 / 658	0.043	1.11e-16	4.44e-15	164 / 505	0.036
Cytokine Signaling in Immune system	50 / 1,107	0.073	1.11e-16	4.44e-15	193 / 725	0.052
Immune System	52 / 2,698	0.178	1.11e-16	4.44e-15	206 / 1,644	0.118
Peptide ligand-binding receptors	13 / 203	0.013	2.53e-12	8.61e-11	10 / 83	0.006
Class A/1 (Rhodopsin-like receptors)	13 / 414	0.027	1.39e-08	4.16e-07	10 / 185	0.013
GPCR ligand binding	13 / 608	0.04	1.12e-06	3.01e-05	10 / 217	0.016
CLEC7A/inflammasome pathway	3 / 8	5.28e-04	5.72e-06	1.37e-04	2 / 4	2.87e-04
TNFs bind their physiological receptors	4 / 30	0.002	8.36e-06	1.84e-04	2 / 13	9.33e-04

Table S4: Demonstration of the top 10 relevant pathway results of Aβ 1-42 treatment.

The following table shows the 10 most relevant pathway results of α-synuclein overexpression and Aβ 1-42 treatment sorted by p-value (Figure 6F)

https://reactome.org/PathwayBrowser/#/ANALYSIS=MjAyMjA3MjkyMDE2MjdfMjIwNDI%3D

Pathway name	Entities				Reactions	
	found	ratio	p-value	FDR*	found	ratio
Interleukin-10 signaling	32 / 86	0.006	1.11e-16	4.66e-15	12 / 15	0.001
Interleukin-4 and Interleukin-13 signaling	27 / 211	0.014	1.11e-16	4.66e-15	29 / 47	0.003
Chemokine receptors bind chemokines	12 / 57	0.004	1.11e-16	4.66e-15	8 / 19	0.001
Signaling by Interleukins	52 / 658	0.043	1.11e-16	4.66e-15	189 / 505	0.036
Cytokine Signaling in Immune system	53 / 1,107	0.073	1.11e-16	4.66e-15	215 / 725	0.052
Immune System	55 / 2,698	0.178	1.11e-16	4.66e-15	228 / 1,644	0.118
Peptide ligand-binding receptors	13 / 203	0.013	1.53e-11	5.52e-10	10 / 83	0.006
Class A/1 (Rhodopsin-like receptors)	13 / 414	0.027	7.47e-08	2.31e-06	10 / 185	0.013
GPCR ligand binding	13 / 608	0.04	5.39e-06	1.35e-04	10 / 217	0.016
G alpha (i) signalling events	11 / 426	0.028	5.42e-06	1.35e-04	3 / 74	0.005

Table S5: Demonstration of the top 10 relevant pathway results of α-synuclein overexpression and Aβ 1-42 treatment.
